# A Deep Learning Approach for Pixel-Level Material Classification via Hyperspectral Imaging

**DOI:** 10.3390/jimaging12060267

**Published:** 2026-06-18

**Authors:** Savvas Sifnaios, George Arvanitakis, Fotios K. Konstantinidis, Georgios Tsimiklis, Angelos Amditis, Panayiotis Frangos

**Affiliations:** 1 Institute of Communication and Computer Systems, National Technical University of Athens, 9 Iroon Polytechniou Str., GR-157 73 Athens, Greece; fotios.konstantinidis@iccs.gr (F.K.K.); georgios.tsimiklis@iccs.gr (G.T.); a.amditis@iccs.gr (A.A.); 2 School of Electrical and Computing Engineering, National Technical University of Athens, 9 Iroon Polytechniou Str., GR-157 73 Athens, Greece; pfrangos@central.ntua.gr; 3 Technology Innovation Institute, Yas Island, Abu Dhabi P.O. Box 9639, United Arab Emirates; arvanitakisgeorge@gmail.com

**Keywords:** hyperspectral imaging, deep learning, material classification, pixel-level classification, real-time object detection

## Abstract

Recent advancements in computer vision, particularly in detection, segmentation, and classification, have significantly impacted various domains. However, these advancements are still strongly tied to RGB-based systems, which are insufficient for applications in industries such as waste sorting, pharmaceuticals, and defence, where material characterization beyond shape or visible colour is necessary. Hyperspectral (HS) imaging captures spatial and spectral information for each pixel and therefore offers a promising route for material-level classification. This study evaluates the potential of combining HS imaging with deep learning for plastic material classification. The work includes: (i) the design of an experimental setup with a HS line-scan camera, conveyor, and controlled illumination; (ii) the construction of an object-disjoint dataset of HDPE, PET, PP, and PS samples with semi-automated mask generation and Raman spectroscopy-based labelling; and (iii) the development of P1CH, a lightweight pixel-wise 1D convolutional hyperspectral classifier. On object-disjoint test images, P1CH achieved 97.44% all-pixel accuracy. A boundary sensitivity analysis, reported separately because semi-automated labels are uncertain at material/background interfaces, yielded 99.94% accuracy after excluding a pre-defined two-pixel border band. Additional ablation, baseline, and robustness analyses show that the proposed pixel-wise spectral approach is effective for small fragments, visually similar plastics, and overlapping materials, while black or very dark plastics remain challenging under the present camera and illumination configuration.

## 1. Introduction

The field of material classification has evolved significantly over the past few decades, transitioning from traditional techniques to the application of deep learning methodologies. Traditional methods, such as thresholding, edge detection, and classical machine learning algorithms (e.g., k-nearest neighbours, support vector machines), rely heavily on manually crafted features and heuristic rules. These methods are often effective for simple tasks but struggle with complex and fine-grained material differentiation due to their inherent limitations in capturing shape and colour variations.

Image classification and segmentation have undergone a paradigm shift with the introduction of deep learning, especially convolutional neural networks (CNNs). Material classification tasks have improved in accuracy and robustness through deep learning models that automatically learn hierarchical features from raw data. However, despite these advancements, several challenges remain when using conventional RGB images, especially in scenarios where the colour or the shape of the object is not that informative regarding its material.

**Figure 1:**
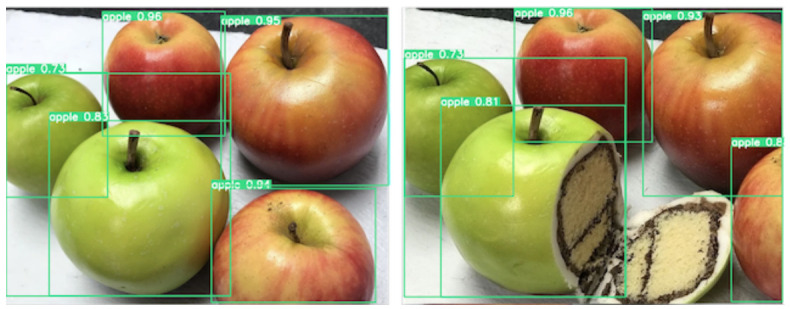
Example of RGB-based object misclassifications. This image depicts pastry cakes that reassemble apples, leading the RGB-based model to mistakenly classify the instances as apples.

RGB images solely rely on spatial features, such as edges, textures, and colours visible to the human eye. This dependency often leads to misclassifications when objects of different classes have similar appearances, as shown in Figure 1. For instance, in the sorting industry, accurately distinguishing between materials with subtle colour differences or complex shapes is crucial for efficiency and quality control. In the pharmaceutical industry, ensuring the purity and correct identification of compounds necessitates fine segmentation capabilities [1]. Similarly, in agriculture, RGB imaging often falls short in detecting diseases in crops that present subtle spectral variations which are not visible in the RGB spectrum [2]. In defence applications, accurate material classification can aid in the detection and identification of hazardous substances, ensuring safety and operational efficiency [3].

Hyperspectral (HS) imaging has emerged as a promising solution to these challenges. Unlike traditional RGB imaging, hyperspectral cameras capture a wide spectrum of light beyond the visible range, providing detailed spectral information for each pixel in an image [4]. This richness in spectral data allows for more precise material classification and segmentation, overcoming the limitations of traditional methods.

Despite its potential, research on computer vision beyond RGB imaging remains limited. The main obstacles are the lack of **publicly available industrial HS datasets**, the cost of **specialised sensors**, the difficulty of obtaining reliable **pixel-level labels**, and the **computational burden** of processing hundreds of spectral bands in real time. In this paper, we propose P1CH (Pixel-wise 1D Convolutional Hyperspectral classifier), a lightweight spectral model designed for pixel-wise material classification of line-scan HS images in a conveyor-belt setting. We emphasize that P1CH does not introduce a new convolutional operator; instead, its contribution lies in a practical and reproducible integration of pixel-wise spectral learning, object-disjoint material validation, low-latency inference, and simplified radiometric normalization for industrial sorting scenarios. The contributions of this work are summarized as follows:

- **Dataset and labelling protocol:** We construct an object-disjoint HS dataset for supervised pixel-level learning, containing HDPE, PET, PP, and PS samples with semi-automated masks and Raman spectroscopy-based material labels.
- **Pixel-wise spectral model:** We design and evaluate P1CH, a compact, real-time 1D CNN that accurately classifies each 224-band pixel spectrum independently, thereby preserving material boundaries and avoiding the boundary smoothing that can occur when large spatial kernels mix spectra from adjacent materials.
- **Quantitative validation:** We evaluate the model on unseen objects and challenging test scenes, including shredded fragments, visually similar plastics, and overlapping materials, and report class-wise precision, recall, F1-score, ablation results, cross-validation, robustness tests, and inference speed.
- **Calibration-aware deployment:** We study a cost-efficient normalization approximation and explicitly compare it with white-reference normalization while discussing its limitations and possible bias under changing illumination or sensor conditions.
- **Limitations and future directions:** We analyse failure modes, especially black plastics, and discuss extensions involving hybrid spatial–spectral models, wider spectral ranges, active illumination, and advanced spectral signal processing.

The rest of the paper is structured as follows: Section 2 describes the related work, while Section 3 provides a high-level description of the system setup and the hardware used in this work. The creation of HS dataset, the spectral preprocessing methodologies along with the semi-automated AI-assisted mask generation are presented in Section 4. Section 5 introduces the architecture of the proposed model, the preparation of the training instances. The training process, along with the selected hyperparameters and the initial results are also discussed in that section. In addition, the empirical performance of P1CH on various challenging scenarios, i.e. mixed materials, shredded objects and overlapping objects, and the performance of the proposed system are discussed in Section 6. This section also explores the limitations of the hyperspectral model in the case of dark-coloured or black objects. Finally, conclusions are drawn and a discussion about future research avenues is provided in Section 7.

## 2. Related Work

Material classification has historically relied on several approaches that span across many domains, including electro-mechanical and chemical analysis techniques. Raman and near-infrared spectroscopy, coupled with multivariate analysis, have widely been used to identify the materials’ composition [5,6,7]. Moreover, the utilisation of X-Ray Diffraction (XRD), Energy-Dispersive X-ray Spectroscopy (EDS) and Atomic Absorption Spectroscopy (AAS) techniques has been proposed because of their high sensitivity and accuracy in detecting microstructural features and hence identifying the elemental composition [8]. Zhang et al. [9] highlighted the use of Differential Scanning Calorimetry (DSC) and Thermogravimetric Analysis (TGA) in accurately determining the thermal properties of various materials. Zhang and Shao [10] emphasized the role of optical microscopy in material.

Recent work in Raman spectroscopy is also relevant because it addresses high-dimensional spectral interpretation, denoising, and reproducibility. Ranasinghe et al. reviewed the role of Raman spectroscopy and liquid biopsy for molecularly specific diagnosis of brain disorders, emphasizing the value of spectral fingerprints for complex biological samples [11]. Wang et al. introduced peak-sensitive logistic regression with elastic-net regularization (PSE-LR), showing that interpretable machine learning models can identify subtle and class-specific spectral peaks in optical spectroscopy [12]. More recently, Wang et al. demonstrated machine learning-enhanced hyperspectral Raman imaging for label-free molecular mapping [13], while Ranasinghe et al. showed that two-dimensional materials can improve signal-to-noise management in surface-enhanced Raman spectroscopy [14]. In addition, RamanSPy provides an open-source framework for standardized Raman preprocessing, analysis, and visualization [15]. Although HS imaging differs from Raman spectroscopy in spectral resolution and physical contrast mechanism, these studies motivate future integration of interpretable spectral feature selection, denoising, and reproducible spectral workflows into HS material classification pipelines.

In an attempt to further improve the performance and robustness of material classification pipelines, various studies explored the potential of deep learning. Weiss et al. in [16] introduce a deep convolutional neural network (CNN) to classify 60 GHz radar data with an accuracy of 97%. Deep neural networks have also been utilised to analyse surface haptic data [17], achieving a precision of 94% and recall of 92%. Moreover, CNNs have been proposed for the case of visual RGB images [18,19,20,21] in various use cases, e.g., commercial waste, steel products, etc. In another work, Konstantinids et al. [22] proposed a multi-modal deep classifier based on ResNet-18 that jointly extracts information from RGB and multispectral cameras to classify plastic polymers, as well as wood by products with an object accuracy of 96%.

While the cited work demonstrates impressive classification performance, these methods are often slow and computationally intensive. HS imaging has become a powerful means for in situ material classification, since it offers the advantage of real-time and efficient data processing. Shaikh and Thörnberg [23] investigated the impact of water vapour on polymer identification by means of short-wave infrared HS imaging yielding an accuracy rate around 88%. According to Shaban [24], HS imaging has been used to determine different characteristics pertaining to concrete without disturbing its structure at sites, where it would not be possible to take samples back to laboratories for analysis; the accuracy is 90%. With 93% accuracy, Capobianco et al. [25] have characterized ancient Roman wall paintings using HS imaging, as well as aiding in the authentication of artwork (Polak et al. [26]), achieving a precision of 95%.

The combination of deep learning and HS imaging has shown great promise in improving the material classification capability in Earth Observation applications. Notable examples include models, e.g., Xception-based, CNN-based systems, and R-VCANet achieving accuracies up to 99%, with precision and recall values around 94% and 93%, and F1 score reaching 91% [27,28,29,30,31]. Venkatesan et al. [32] applied deep recurrent neural networks in medical HS images to achieve feature recognition with a precision rate of about 96%. Xiong et al. [33] developed material tracking methods for HS videos using deep learning, achieving an F1 score of 94%. Medus et al. [34] applied CNNs to classify HS images in industrial food packaging, reporting an accuracy of 99%. Okada et al. followed a patch-wise approach for the identification of 5 different mineral types, using a VGG16-based CNN to classify the acquired HS images they achieved high accuracy over 90% [35]. Extreme Learning combined with Stacked Autoencoders for feature extraction has also been applied on HS images, in [36], for the detection of plastic films within cotton feed stock, creating a pixel-level classification map with accuracy up to 95%. Zhu et al. in [37], also attempted to classify different cotton seeds. Their work encompasses a multivariate analysis for manual extraction of the 10 most informative features, which were subsequently fed to CNN for the classification task, resulting in a prediction accuracy of 88%. Moreover, Artzai et al. worked with HS images with 76 bands of non-ferrous metals, and utilised them to train a CNN-based U-Net-like network [38]. This approach analysed the HS images as a whole and created a classification map with an accuracy equal to 95%. Finally, Roy et al. [39] proposed a methodology that incorporates PCA as a preprocessing step with a deep convolutional network, i.e., HybridSN, for the pixel-wise classification of HS images, achieving an accuracy score of 99%.

The aforementioned HS classification studies have yielded impressive results. However, many models either process HS images as complete spatial patches or use 2D/3D kernels that combine neighbouring pixels. This spatial context can be beneficial in homogeneous regions, but it may also mix spectra at material/background interfaces or at boundaries between overlapping objects. The proposed P1CH model intentionally uses a pixel-wise 1D spectral architecture to prioritize boundary preservation and material-specific spectral signatures. This design choice also has limitations: it ignores potentially useful spatial context. Therefore, an important future direction is a hybrid spectral–spatial architecture that adds limited, edge-aware spatial information without smoothing spectra across material boundaries.

## 3. Hyperspectral Imaging Experimental Setup

This section provides a detailed description of the physical infrastructure of the system where the dataset was created using materials that were scanned by the RGB and HS sensors.

### 3.1. Imaging Spectroscopy

HS imaging integrates conventional imaging and spectroscopy to simultaneously capture spatial and spectral information across a wide range of wavelengths. This pixel-level data enables precise object localization, material classification, etc. [40]. To this end, a variety of sensors have been used. In particular, push-broom sensors capture spectral information across a swath as the sensor moves, line scan sensors capture data one line at a time, while whiskbroom sensors scan point-by-point to build an image, and snapshot sensors capture the entire spectral image in a single exposure.

### 3.2. Camera

The SPECIM FX17 line scan camera (Specim, Spectral Imaging Ltd., Oulu, Finland), following push broom technology, utilises a matrix detector and an imaging spectrograph to capture spectral data efficiently. Light enters through high-performance optics and an entrance slit, forming a line image that the spectrograph disperses into a spectrum (900–1700 nm, across 224 bands). This setup allows each axis of the detector to record spatial position and spectral information simultaneously. It ensures measurement stability despite object or camera movement, requires less illumination power while achieving higher intensity, and is significantly more efficient than filter-based cameras, yielding a purer spectrum.

### 3.3. Conveyor

Once the line scan camera is selected for capturing, the system comprises a conveyor belt that facilitates the horizontal movement of objects at an adjustable speed, in order to achieve synchronised sampling frequency of the HS sensor and objects’ movements. The camera was placed 0.73 m from the conveyor, while the illumination source was placed 0.5 m vertically above the conveyor belt, at a 45-degree angle, and 0.3 m horizontally from the centre of the camera.

### 3.4. Illumination

The deployed, custom-made LDL-222X42CIR Full-Spectrum bar light manufactured by CCS Inc. (Sint-Pieters-Leeuw, Belgium), offers better performance for challenging imaging spectroscopy scenarios in contrast with LED lights [41]. Specifically, it includes four different halogen bulbs that emit light in distinct regions of the spectrum and a power rating of 87 W. This allows it to span the complete range of wavelengths from 400 nm to 2400 nm. This light source contains a dispersion layer that uniformly distributes the output light in multiple directions, resulting in more consistent illumination of the scene and the objects within it.

For each training acquisition, the same halogen illumination geometry was maintained, but the second scan was acquired after small changes in exposure/lamp output and object placement. The resulting spectral intensity variation was moderate rather than exhaustive; therefore, these duplicate scans should be interpreted as acquisition augmentation under controlled laboratory illumination, not as a complete validation under all possible industrial lighting conditions. A dedicated robustness analysis with multiplicative intensity perturbation and additive spectral noise is reported in Section 6.

### 3.5. Acquired Data

The HS camera employed in this work is ample for achieving a maximum sampling rate of 400 FPS, with each scanned line being of shape 640×224 (pixels×bands). Moreover, the camera provides the option of applying on-chip spatial and/or spectral binning, hence reducing the respective dimensions by factors of ×2, ×4, or ×8. In this work, no spectral or spatial binning was applied; thus, the acquired data were of shape $n_{rows}\times 640\times 224$, where $n_{rows}$ depends on the selected sampling rate and the acquisition duration. Finally, it is worth mentioning that the pixel size of the selected camera is 0.9375 mm, thus allowing for very precise and accurate segmentation.

## 4. Dataset

In this section, the dataset collection procedure is described. First, we will provide an overview of the plastic samples (i.e., the list of distinct materials and their counts) that were scanned by the HS camera. The data preprocessing pipeline is then described, which converts raw images into a format compatible with deep learning frameworks. Finally, an extensive discussion on the semi-automated AI-assisted labelling employed in this work to annotate images is provided.

### 4.1. Dataset Description

**Remark 1.**  *To ensure clarity and consistency, we define the following terminology that will be used throughout the remainder of this paper. The term **object** refers to the physical object. The term **sample** refers to a pair (X,y), where X is a feature vector of size 1×224 and y is an integer value denoting the known target class. The term **class** refers to the material categories of interest in the HS dataset (i.e., HDPE, PET, PP, and PS).*

For the generation of the train and test datasets, an ensemble of plastic objects from four polymer categories was collected: *HDPE*, *PET*, *PP*, and *PS*. The split was performed at the physical object level. No physical object used for training was used for testing. This object-disjoint protocol is important because a random pixel-level split across the same objects would overestimate generalization.

**Training set:** In total, 169 physical objects were selected to represent the four classes. Each object was captured twice under slightly different controlled illumination/exposure conditions, resulting in eight training HS images (two per class). Each training image contains objects from a single material class. This design was chosen to simplify accurate object-level mask generation and to avoid ambiguity during Raman-based material assignment. During learning, however, the model receives individual spectral vectors of size 1×224 and does not receive the image identity, object shape, object size, or neighbourhood context as input. Therefore, the model is encouraged to learn class-specific spectral signatures rather than object layouts. A breakdown of the dataset composition is presented in Table 1, while Table 2 summarizes the type, colour and shape distributions of the objects selected for this dataset.

At this point, it is important to mention that a 5th class was introduced to the dataset. Namely, this class is *Background* and it represents the set of pixels depicting the conveyor belt’s surface. Since the conveyor belt is apparent in every HS image, no matter the type of plastic, initially the number of background pixels was ×10 higher than any other class, potentially leading the model to be biased in favour of this class. To handle this, a random subset of background pixels was selected, ensuring its size will be equal to the maximum number of pixels of the plastic classes, i.e., 831,340 pixels.

**Test set:** Eighty additional objects, which were not used during training, were selected for testing. The test set was intentionally designed to be more demanding than the training set and includes (i) unmodified objects similar to industrial waste items, (ii) small shredded fragments with irregular shapes, and (iii) mixed or overlapping objects where different materials touch or partially occlude each other. This discrepancy between single-class training images and mixed-object test images is deliberate and aims to evaluate whether spectral material signatures learned from clean object masks transfer to realistic sorting scenes.

Furthermore, according to the literature [42,43], HS cameras (up to 1700 nm) cannot capture information regarding plastics that are coloured black. In order to examine that claim, a 3rd black-plastics dataset was generated and it is presented in the Table 3.

Throughout the literature, it is strongly advised to (a) use the spectral calibration matrix (specifically provided by the camera manufacturer) [44] and then (b) normalize the calibrated data using White and Black reference.

**White reference:** A white reflection target is required with the key property of reflecting the incident radiation uniformly across all wavelengths. In this manner, the true maximum pixel value a HS camera can capture, given the illumination source, is calculated and ultimately creates the white reference. It should be mentioned, though, that white reflection targets are often not commercially available and their cost grows exponentially with respect to its dimensions.

**Black reference:** On the other hand, the black reference is utilised to model the sensor’s electronic noise, caused by the electrons’ random movement due to the sensors’ temperature.

By modelling that noise, one can subtract the black reference both from the HS image and the white reference to acquire the noise-corrected version of both images. A mathematical formulation of the black-white reference normalization is provided below:

(1) $$I_{\mathrm{norm}}\left(x,y,\lambda \right)=\frac{I\left(x,y,\lambda \right)-I_{\mathrm{black}}\left(\lambda \right)}{I_{\mathrm{white}}\left(\lambda \right)-I_{\mathrm{black}}\left(\lambda \right)},$$

where $I_{\mathrm{norm}}\left(x,y,\lambda \right)$ is the normalized HS image at pixel (x,y) and wavelength λ, I(x,y,λ) is the raw HS image at pixel (x,y) and wavelength λ, $I_{\mathrm{black}}\left(\lambda \right)$ is the black reference image at wavelength λ, $I_{\mathrm{white}}\left(\lambda \right)$ is the white reference image at wavelength λ.

The black reference $I_{\mathrm{black}}\left(\lambda \right)$ was acquired by closing the shutter of the camera and capturing 1000 lines, which were then averaged. For the maximum value the HS camera can capture, an assumption was made that it can be approximated by calculating the maximum pixel value within the train set. An approximation of (1), that does not require an expensive white reflection target, is provided below:

(2) $$I_{\mathrm{norm}}\left(x,y,\lambda \right)=\frac{I\left(x,y,\lambda \right)-I_{\mathrm{black}}\left(\lambda \right)}{M},$$

where $I_{\mathrm{norm}}\left(x,y,\lambda \right)$ is the normalized HS image at pixel (x,y) and wavelength λ, I(x,y,λ) is the raw HS image at pixel (x,y) and wavelength λ, $I_{\mathrm{black}}\left(\lambda \right)$ is the black reference image at wavelength λ, *M* is the dataset’s maximum pixel value.

**Remark 2.**  *The spectral calibration and the normalization as operations are a sequence of matrix multiplications. It seems, based on our experiments, that in controlled acquisition environments, this transformation can be learned directly in the training process. The approximation in (2) is not intended to replace standard radiometric calibration in all HS imaging applications. Rather, it is a practical normalization option for the controlled acquisition configuration used in this work, where the camera, illumination geometry, exposure settings, conveyor background, and material set are fixed.*

### 4.2. Ground Truth Mask Generation

**Figure 2:**
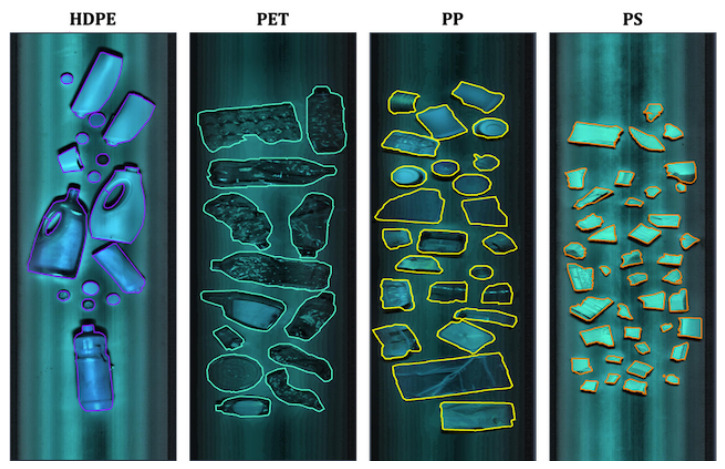
The false-colour contrast stretched HS images used for the generation.

As in every supervised learning application, a set of ground truth labels is needed to ensure successful training of the neural network model. To this end, an AI-assisted methodology was deployed for the generation of the binary masks, presented in Figure 2, which later on will be utilised as training labels.

#### Semi-Automated Segmentation

The first step in the proposed methodology is the creation of a false-colour RGB version of the HS image. To this end, the standard deviation of each channel in the original image was calculated as a measure of its contrast. The three channels with the highest contrast were selected and sorted in ascending order of wavelength, for each image, in order to create the false-colour RGB image. An adaptive histogram stretching algorithm [45] was also applied to the respective RGB versions in order to further increase the contrast and make the objects’ edges as sharp as possible without altering the spatial content of each image.

The false-colour histogram stretched images were subsequently utilised for the generation of segmentation masks. SAM [46] was employed for the semi-automated mask generation task. In detail, positive and negative points were given as prompts to the model in order to generate a first estimation of the mask. The predicted mask was then visually inspected and refined, when needed, aiming for precision maximization at the boundaries of the object. This procedure was repeated for every image and every object depicted within an image of the dataset and the final results can be seen in Figure 2.

### 4.3. Acquisition Pipeline

**Figure 3:**
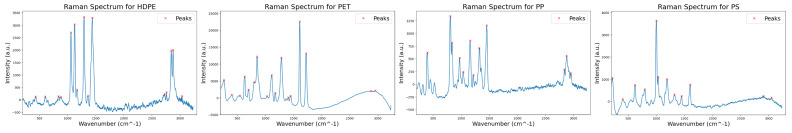
The Raman spectra for the 4 material classes. For the HDPE spectrum, the peaks at 1058 cm^−1^, 1123 cm^−1^, 1291 cm^−1^, 1437 cm^−1^, and the range from 2843–2876 cm^−1^ are characteristic of high-density polyethylene (HDPE). These peaks correspond to the vibrations of the molecular structure of HDPE, specifically indicating the various stretching and bending vibrations of the C-H bonds. Accordingly, for the rest of the spectra, it is pointed out that two characteristic peaks at 1607 cm^−1^ and 1721 cm^−1^ were detected in the top-right plot and correspond to the vibrations of the phenyl group in the polyester. Additionally, the peaks in the range of 1100–1200 cm^−1^ indicate the stretching vibrations of the C-O group. The peaks observed at 970 cm^−1^, 1034 cm^−1^, 1360 cm^−1^, 1453 cm^−1^, and 2946 cm^−1^ in the bottom-left plot are associated with the vibrations of the methyl group (CH_3_) in polypropylene, while the intense peak observed at 1010 cm^−1^, in the bottom-right plot, along with the peak at 1598 cm^−1^, suggest the presence of polystyrene.

The final step of the ground truth generation is to assign a class to each of the aforementioned masks. To this end, Raman spectroscopy [47] was employed. Raman spectroscopy is a powerful analytical technique used to observe vibrational, rotational, and other low-frequency modes in a system. It relies on inelastic scattering, or Raman scattering, of monochromatic light, typically from a laser. When light interacts with molecules, it induces vibrations or other excitations in the system, shifting the energy of the laser up or down. This creates peaks in the acquired spectrum, providing a unique fingerprint by which molecules can be identified [47,48]. In Figure 3, an example of the spectrum for each of the 4 plastic types is presented, where the red marked peaks indicate the existence of each polymer in the under examination plastic object.

In this manner, each object annotated in the previous step was individually scanned with the Raman equipment and its spectrum was analysed in order to identify the indicative peaks for each class. The results of the Raman spectroscopy analysis were used as the class of each of the aforementioned masks.

## 5. P1CH: Architecture, Training & Inference Pipeline

### 5.1. Architecture

**Figure 4:**
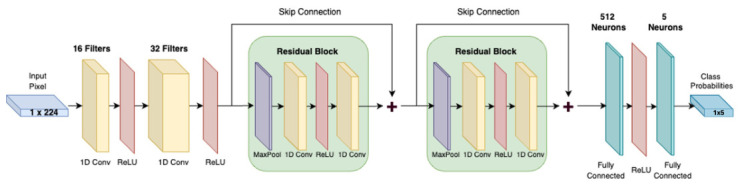
The high-level architecture of the proposed Pixel-wise 1D Convolutional Hyperspectral (P1CH) classifier.

The proposed HS image classification model uses a **1D CNN architecture** to capture and process spectral information. The architecture uses the following building blocks (a) 2 convolutional layers, (b) 2 residual blocks, and (c) 2 fully connected layers to accurately classify HS data. The architecture of the P1CH classifier is shown in Figure 4.

**Convolutions:** The input pixel of shape (1×224) is passed through an initial convolutional layer with 16 size-3 filters and padding to preserve dimensions. Afterwards, a 32-filter, 3×3 convolutional layer with padding follows. After each convolutional layer, a ReLU activation function introduces non-linearity and a max-pooling layer, with both kernel size and stride set to 2, that reduces data dimensionality.

**Residuals:** The model uses two residual blocks for feature extraction and learning. The first residual block receives the output from the second convolutional layer and processes it through two convolutional layers with 64 filters each, maintaining a kernel size of 3. Batch normalization is also applied after each convolutional layer. This output is added to the block’s input—through a skip connection—and passed though a ReLU activation function. The second residual block follows a similar structure, but with 128 filters in each layer.

**Fully Connected:** The processed features are flattened and passed through a fully connected layer with 512 neurons, followed by a dropout layer. A second fully connected layer follows with five neurons, one for each class P1CH is expected to identify (i.e., Background, HDPE, PET, PP, PS). Finally a soft-max layer provides the final classification probabilities.

The use of residual 1D convolutional blocks aims to provide a compact spectral feature extractor that operates directly on each pixel spectrum, avoids mixing neighbouring material spectra at object boundaries and can be executed at line-scan speed (300 lines/s), rather than a new primitive neural-network design. The residual connections were retained because they improved optimization stability for 224-band input spectra while adding limited computational overhead. The architectural ablation in Section 6 quantifies the effect of removing residual blocks and post-processing.

### 5.2. Data Pre-Processing

As mentioned in Section 5.1, the model expects a 1-dimensional vector as input. In this work, the input vectors are the individual pixels of the dataset’s images. Up to this point, however, the dataset consists of HS images, hence it is necessary to convert the images into a set of pixels.

To this end, a data handling pipeline was implemented to efficiently utilise the large volume of data encoded within HS images. In particular, a memory-mapped array uses the operating system’s virtual memory capabilities to map a disk file directly into the address space of the application, allowing for efficient, random access to large datasets without loading the entire file into the memory. In this manner, by employing memory-mapped arrays, out-of-core processing is achieved, which significantly reduces the memory footprint and improves the performance of data-loading operations.

By exploiting the capabilities of memory-mapped arrays, the entire set of images, along with their respective ground truth masks, are flattened across the spatial dimensions, thus creating the desired feature vectors, each of size 1×224. The feature vectors are subsequently randomly shuffled and split in two subsets; the train and the validation set with ratios of 90% and 10%, respectively.

### 5.3. Model Training

**Figure 5:**
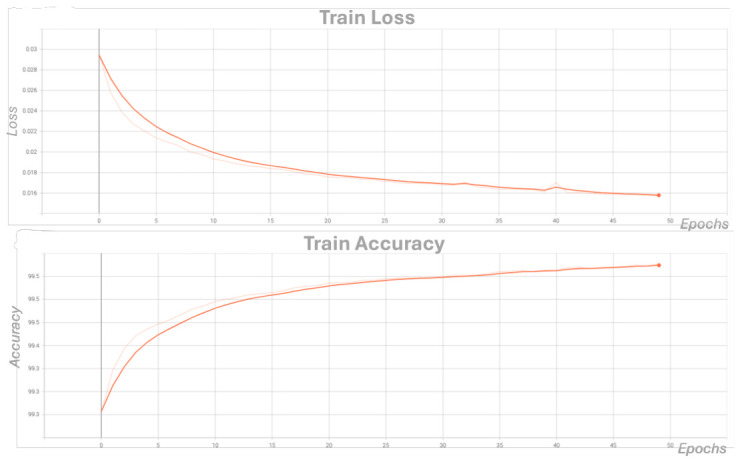
P1CH loss (**top**), and accuracy score (**bottom**) training curves.

**Figure 6:**
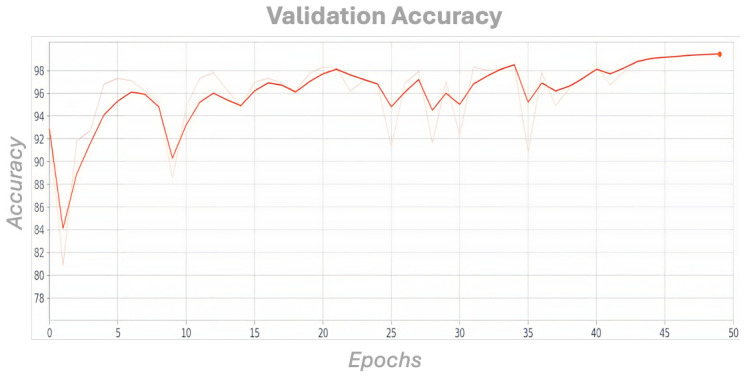
The model’s performance in the validation set, during training, with the best accuracy score achieved in epoch 35.

The model was implemented in Python 3.10 using PyTorch 2.9. Training was performed with the Cross-Entropy loss [49] and the AdamW optimizer ($\beta_{1}=0.9$, $\beta_{2}=0.999$, weight decay $10^{-4}$). The batch size was set to 8192 pixel spectra. The model was trained for 50 epochs using a warm-up cosine learning-rate schedule: the learning rate increased from $10^{-4}$ to $10^{-3}$ during the first 10 epochs and then decreased to $10^{-4}$. Dropout with probability 0.4 was used before the final classification layer. The checkpoint with the highest validation accuracy was retained. Unless otherwise stated, experiments used random seed 42 and were executed on a workstation equipped with an NVIDIA RTX A4000 GPU (16 GB), 64 GB RAM, and an Intel i7-class CPU. The final checkpoint achieved 99.88% training accuracy and 99.45% validation accuracy on the pixel-level validation split as depicted in Figure 5 and Figure 6. The final performance reported in Section 6 is evaluated on object-disjoint test images, which were not used for training, validation, hyperparameter selection, or post-processing tuning.

### 5.4. Post-Processing

P1CH processes each line independently and achieves approximately 300 lines per second for input lines of shape 640×224. For the qualitative figures in this paper, the predicted lines are accumulated into a complete classification map and then post-processed. This accumulation is convenient for offline visualization but is not a strict deployment requirement.

**Median filter:** a kernel of size 5, is applied to the reconstructed classification map. In this manner, misclassifications that reassemble the Salt & Pepper noise are corrected by substituting them with the median value of the surrounding 5×5 region, as described in (3).

(3) $$I^{\prime }\left(x,y\right)=\mathrm{median}\left\{I\left(i,j\right)|\left(i,j\right)\in W\left(x,y\right)\right\},$$

where I(x,y) is the original image, $I^{\prime }\left(x,y\right)$ is the filtered image, and W(x,y) is the window centred at (x,y).

**Morphological opening and closing:** the application of such filters enhances the overall quality of the segmentation by removing small noise artifacts and refining object boundaries. The opening filter effectively eliminates small, isolated regions of misclassified pixels, while the closing filter fills in small gaps and smoothens the contours of classified regions [50,51]. Morphological filters are presented respectively in (4) and (5).

(4) I∘B=(I⊖B)⊕B,

where *I* is the original image, *B* is the structuring element, ⊖ denotes the erosion operation, ⊕ denotes the dilation operation.

(5) I•B=(I⊕B)⊖B,

where *I* is the original image, *B* is the structuring element, ⊕ denotes the dilation operation, ⊖ denotes the erosion operation.

Together, these morphological operations improve the structural integrity of the classification map, leading to more accurate and visually coherent segmentation results.

In a continuous conveyor-belt implementation, the same operations can be applied with a sliding line buffer. A 5×5 median filter requires only two future lines, corresponding to approximately 2/300=6.7 ms of look-ahead at the measured processing rate. A subsequent 3×3 morphological opening/closing operation adds only a small local buffering requirement. Therefore, the post-processing latency is expected to be dominated by the physical camera-to-actuator distance rather than by the filtering operations. To quantify the contribution of post-processing, Section 6 reports an ablation comparing raw P1CH predictions, median filtering, and median filtering followed by morphology.

## 6. Results & Discussion

In this section the results of the proposed work on pixel-level material classification of HS images are presented; the structure of the sections is as follows: (a) overall performance, (b) capacity to analyse randomly shredded objects, (c) ability to distinguish mixed overlapping materials and (d) limitations are discussed.

### 6.1. Overall Performance

**Figure 7:**
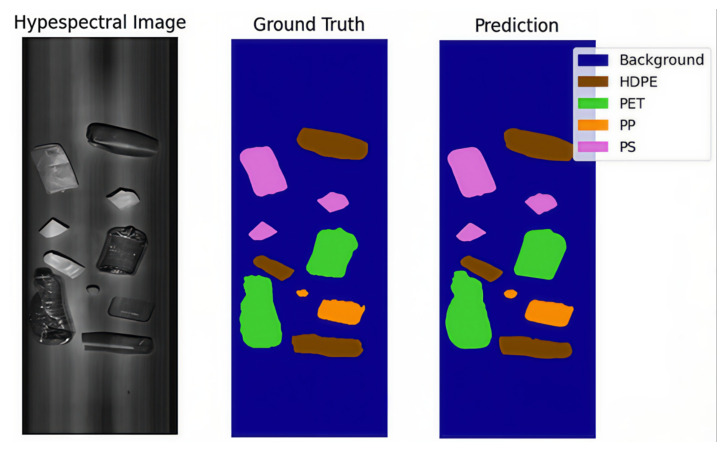
The false-colour version of the HS images, the ground truth mask, as well as the generated classification map from the proposed model.

As discussed in Section 4, the test-set presented in Table 4 includes images of all the HDPE, PET, PP, and PS classes, which have not been utilised in the training subset. In this manner, it is guaranteed that no same object or pixel is simultaneously evident in both train and test data. Figure 7 shows the original image as well as the ground truth and the prediction of each pixel.

**Figure 8:**
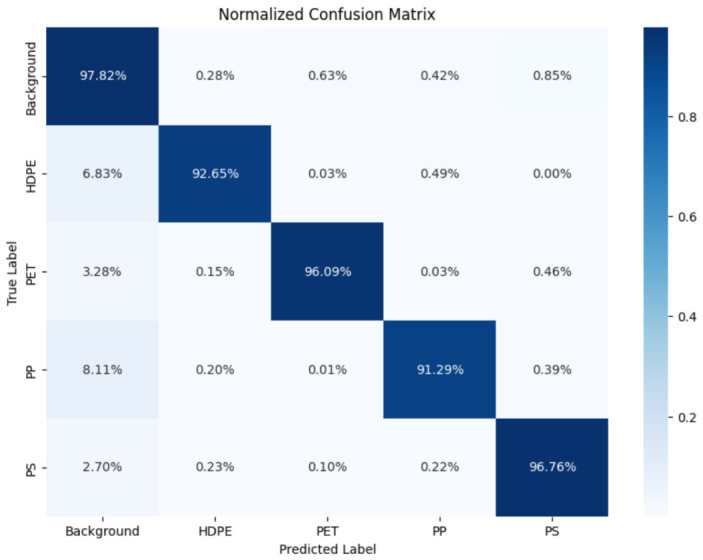
Confusion Matrix describing models performance in classifying materials on pixel-level.

To quantify the model’s performance, the accuracy score was evaluated, at pixel level, for the whole test set. The overall accuracy achieved by the model is **97.44**%. The confusion matrix is presented in Figure 8, summarizing the classification performance among different material classes. The cell values in the confusion matrix are row-wise normalized, i.e., normalized with respect to the total number of samples in each class. Table 5 reports class-wise precision, recall, and F1-score. The primary all-pixel accuracy of the model on the object-disjoint test set is 97.44%. The lower foreground F1-score for PP is mainly caused by boundary disagreements and confusion with background pixels, rather than by systematic PP-to-other-plastic confusion.

In addition to the fixed test set, we performed five-fold object-level cross-validation using the non-black plastic objects. The folds were generated at the object level rather than at the pixel level. Specifically, the original masks were used to identify each object region and extract the pixels belonging to that object. These object-wise pixel groups were then assigned to the cross-validation folds, ensuring that pixels from the same object were not split across different folds.The resulting overall accuracy was 97.98% ± 0.21%, supporting the conclusion that the model is not simply memorizing individual objects. The cross-validation results are consistent with the fixed object-disjoint test accuracy reported above.

To further validate the performance of the proposed P1CH model, the state-of-the-art HybridSN methodology [39,52] was replicated and evaluated on the same test dataset. This approach ensures a direct and fair comparison under identical conditions. The overall accuracy achieved by the HybridSN model on this dataset is significantly lower, at **21.81%**. In contrast, the P1CH model achieves an accuracy of **97.44%**, along with superior recall and Kappa score, as shown in Table 6. It is important to note, however, that HybridSN was originally developed and benchmarked on the Indian Pines dataset—a single-image dataset—where training and validation splits, regardless of method, lead to strong overfitting and limited generalization. Moreover, the hyperspectral scenes HybridSN was originally validated against consisted of local spectral–spatial patches that belong to the same land-cover region. In our conveyor-belt setting, many patches include object/background interfaces or multiple touching materials. This mismatch makes patch-based spectral–spatial learning less suitable unless boundary-aware patch selection is introduced. This limitation was evident in this study as well: when HybridSN was trained on a diverse set of hyperspectral images (the same as those used for training P1CH) and evaluated on separate objects, its performance deteriorated substantially. These findings underscore both the practical advantages and the generalization capability of P1CH in realistic hyperspectral classification tasks.

The performance metrics summarized in Table 6 further underscore the effectiveness of the proposed P1CH classifier. Notably, the accuracy achieved by P1CH (97.44%) is significantly higher than that of the HybridSN model (21.81%), highlighting its superior capacity to classify pixels across the test set. Moreover, the recall value of P1CH (94.92%) demonstrates its robustness in identifying material classes with high consistency, in stark contrast to the HybridSN model, which achieved a recall of only 7.86%. The Kappa score further illustrates the reliability of the P1CH classifier, with a value of 0.9295 compared to the negative score produced by HybridSN, reflecting its struggles with pixel-level classification tasks.

Beyond classification accuracy, the computational efficiency of P1CH is an important advantage. P1CH achieves an inference speed of **300 lines per second (2.06 s per image)**, far surpassing HybridSN’s **29 lines per second (34.29 s per image)**. This substantial difference not only demonstrates the lightweight design of P1CH but also makes it a practical choice for almost real-time applications, where rapid and reliable pixel-level classification is crucial. With these results, P1CH emerges as a robust, accurate, and highly efficient solution for HS image pixel-wise classification in demanding environments.

**Figure 9:**
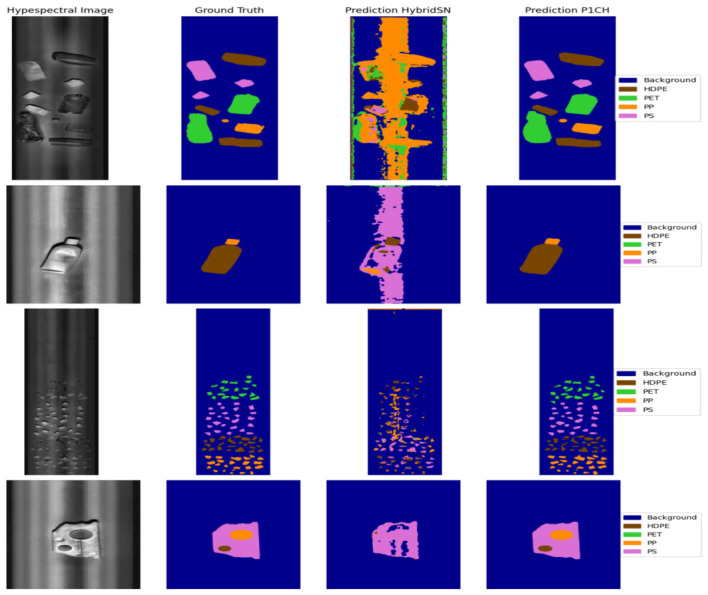
From left to right: The false-colour HS image, the Ground Truth mask, the classification maps generated by HybridSN model, as well as the generated classification map, by the P1CH classifier, in the aforementioned scenarios.

A visual comparison of predictions by both models, on the test set, is presented in Figure 9. The HybridSN model struggles with pixel-level classification accuracy, particularly at object boundaries, and often misclassifies a large portion of pixels. In contrast, the P1CH model demonstrates precise segmentation and classification, even in challenging scenarios involving overlapping objects or small irregular shapes. These findings validate the effectiveness of the P1CH model and its potential applicability in real-world HS imaging tasks.

### 6.2. Boundary Sensitivity Analysis

**Figure 10:**
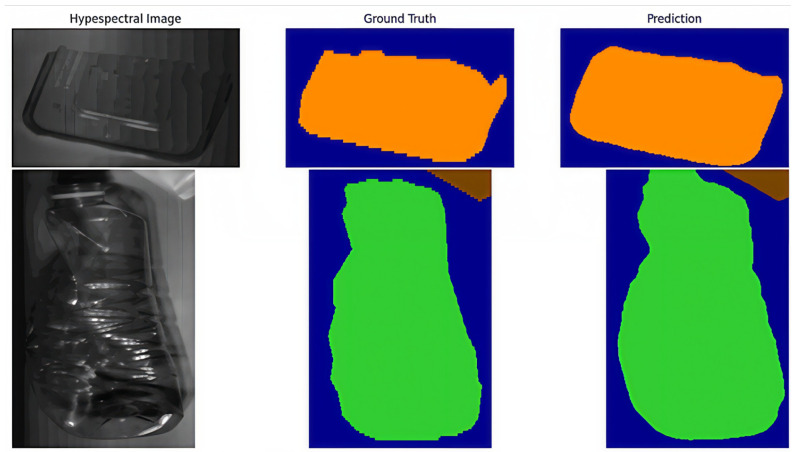
Zoomed-in crops. In the first row the bottom-right PP object of Figure 7 is depicted with the GT and Predicted masks. In the second row the lower-left PET object is presented.

Moreover, it can be calculated that 97.45% of the error comes in the borders between objects and the background, as suggested by the relatively low recall score. After more careful inspection, it is not straightforward to conclude that the error is actually on the predictions of P1CH and not on the ground truth masks. Figure 10 presents two zoomed-in crops of Figure 7, where it is clearly shown that in both cases P1CH has the capacity to generate much smoother and precise masks for the respective object while simultaneously correctly predicting the material class. By comparing the zoomed crops, one can also realize the mistake made on the top right side of the PP object’s mask, as well as at the PET bottle’s spout. In both cases, P1CH was capable of predicting a more precise than the semi-automatically generated mask.

For the boundary sensitivity analysis, a band of border pixels was defined before evaluation as the morphological gradient of the ground-truth foreground mask dilated by a disk/diamond structuring element with radius two pixels. This creates a fixed band around material/background interfaces and around touching object boundaries. The band was not selected from the model errors and was not used for training or post-processing. When this pre-defined boundary band is excluded, the error rate decreases to 0.0653%, corresponding to 99.94% accuracy.

### 6.3. Shredded Materials

Beyond overall accuracy metrics, it is crucial to evaluate the model’s effectiveness in classifying irregularly shaped plastic objects. These shapes, which often pose challenges for traditional RGB-based computer vision models, frequently appear in real-world applications. In this manner, an ensemble of plastic objects was collected and shredded in small, irregular pieces, and then placed on the conveyor belt. P1CH classifier demonstrates remarkable capabilities in classifying on the pixel level the different material classes, clearly exemplifying its superior performance compared to traditional RGB-based instance segmentation algorithms.

Traditional RGB models often struggle with such high variability due to their reliance on surface-level features like colour, texture and shape, which are limited in the case of small shredded objects. On the contrary, P1CH is trained to purely exploit the rich spectral content of each pixel in the HS image, hence successfully tackling the challenge of small and irregularly shaped objects. Another key advantage of the proposed model is its resilience to noise and artifacts commonly found in RGB images. While traditional models can be easily misled by variations in lighting and surface texture, the spectral information utilised by the proposed model provides a more stable basis for classification.

**Figure 11:**
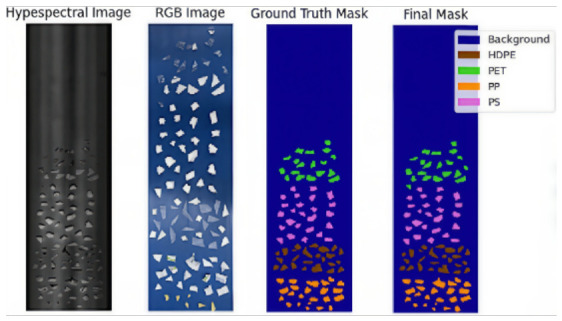
From left to right: The false-colour HS image, the RGB equivalent image, the Ground Truth mask, as well as the classification map generated by the P1CH classifier, in a challenging, cluttered scene, where the objects are small with irregular shapes and similar textures.

In Figure 11 the HS and the respective RGB images of the shredded plastic objects of HDPE, PET, PP and PS are depicted. Along with the aforementioned images, the Ground Truth and the Predicted masks are presented in the same figure. The achieved accuracy in this specific image is 98.9%, with all the misclassifications falling under the borders case described in the previous section.

Visual comparison between the ground truth and the predicted mask indicates a high level of agreement in the classification of the plastic fragments. Each class—HDPE, PET, PP, and PS—is distinctly identified and accurately located in the predicted mask. In detail, even though the objects have similar colours regardless of their class, being either white or transparent, the model was able to correctly classify all the objects to their respective category, as indicated in the ground truth mask. This result underscores the robustness of P1CH is **invariant to colour and size** of the objects by solely relying on the spectral content of each individual pixel.

**Figure 12:**
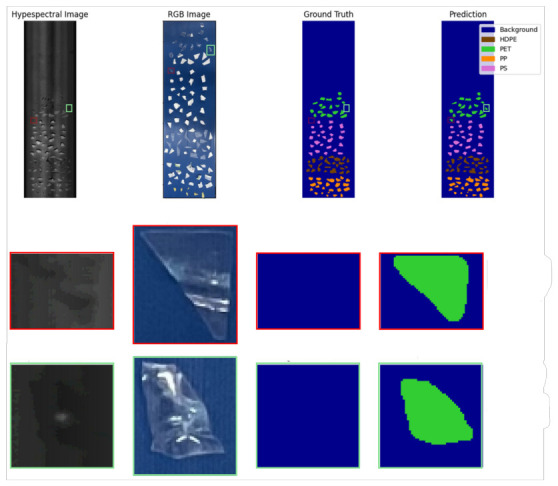
Zoomed-in crops of Figure 11, highlighting two small PET shards that were mistakenly omitted from the labelling process (denoted with red and green boxes), and yet were detected by the model.

Even though the points just presented are important, the most impressive observation from this experiment is the model’s ability to detect objects that were not even visually identified during the labelling process of the HS images. Figure 12 specifies two regions in the HS image, denoted with red and green boxes respectively, in which there are two small shards of PET. Inspecting the zoomed-in crops, it is almost impossible to detect those objects, yet the proposed HS approach clearly identified these two objects as PET. This outcome is only possible through the analytical processing of the spectral information of pixels in those regions, resulting in very precise masks and ultimately correctly classified objects.

### 6.4. Mixed Overlapping Materials

**Figure 13:**
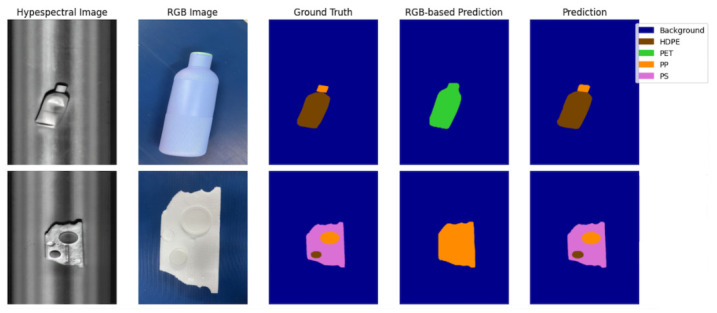
From left to right: The false-colour HS image, the RGB equivalent image, the Ground Truth mask, the classification maps generated by an RGB-based model using the RGB equivalent images, as well as the generated classification map, by the P1CH classifier, in the scenario of mixed or overlapping materials.

In addition to the challenge posed by small irregularly shaped objects, conventional RGB-based detection models often fail to accurately identify distinct objects that are either attached to one another or overlapping. In this subsection, an analysis is conducted to evaluate the model’s capacity to segment objects of different class, while accurately classifying each individual pixel to its respective class. To this end, two experiments were carried out, and in Figure 13 the HS and RGB images and the respective masks of the utilised objects are depicted. To further highlight model’s ability to classify materials and segment their instances within an image, a fine-tuned (on the specific classes RGB-based instance segmentation) model was also employed to segment the RGB images. The predictions of the RGB-based model are depicted in the 4th column of Figure 13.

In the first experiment, a common commercially available light-blue shampoo bottle was selected. From a visual point of view, as seen in the RGB image, the body of the shampoo container and its lid appear almost identical, making it impossible, even for a human, to realize that those two parts are different types of plastic, i.e., HDPE and PP. From the first row in Figure 13, it is immediately obvious that the RGB-based model was able to identify the contours of more complex objects, but not the individual parts, i.e., body and lid. Moreover, the RGB-based model failed to predict the class of both samples, assigning them to the PET class. Finally, due to shadows and non-uniform illumination of the scene, the RGB-generated mask lacks precision as it includes pixels from the background too. On the contrary, the proposed P1CH classifier not only generated highly precise masks for both the body and the lid, but also proved capable of classifying those two parts in their respective class. The overall accuracy of the prediction for this specific test case is 98.8%, with disagreements in a pixel’s class being evident only between background and not the material classes.

In the second experiment, the case of overlapping materials with a very similar (white) colour was examined. To this end, a white PS flat surface was selected, on top of which a white PP lid (top right) and a white HDPE lid (bottom left) were placed. The respective masks and images are depicted in the second row of Figure 13. Looking at the RGB image, no distinctive textures can be detected for the two lids, while some minor changes in texture may be identified between the lids and the PS surface. Therefore, given the uniformity in colour and the very low variance in texture, as anticipated, the RGB-based model failed to detect all three objects apparent in the scene. Once again, the generated mask contains more complex objects with no regard for the two lids. Moreover, the class prediction of the RGB-based is inaccurate since it considers the PS surface as PP. In contrast to the RGB case, the proposed HS model effectively utilises the rich spectral signature encoded in each pixel being able to precisely segment all three components of the image, while classifying materials on pixel-level with 99.54% accuracy.

The results presented above underscore a pivotal advancement in material classification and segmentation capabilities. The most remarkable achievement demonstrated here is the proposed HS model’s ability to accurately identify and classify distinct, overlapping objects with complex boundaries. This marks a significant breakthrough, as conventional RGB-based models consistently fail under these conditions, misidentifying materials and producing imprecise masks. The superior performance of P1CH classifier achieving up to 99.54% accuracy even in the presence of overlapping objects, illustrates a transformative improvement in HS imaging applications, paving the way for more sophisticated and reliable material detection and sorting systems in real-world environments.

### 6.5. Ablation Studies

#### 6.5.1. White-Reference Approximation

To evaluate the effect of the proposed white-reference approximation, we compared three preprocessing variants: (i) manufacturer-style black/white reference normalization, (ii) the proposed black-reference plus training-maximum normalization, and (iii) black-reference correction without maximum scaling. The comparison is reported in Table 7.

The small difference between the first two rows indicates that the proposed approximation is adequate for the present controlled setup. Nevertheless, the approximation may introduce bias when a new scene contains pixels brighter than those observed during training, when the lamp spectrum or exposure changes, when the sensor response drifts, or when highly reflective/dark materials outside the training distribution are introduced. In such cases, a white reference, per-band percentile normalization, periodic recalibration, or domain-adaptation strategy should be used. Therefore, the revised interpretation is that P1CH is tolerant to the simplified normalization under the acquisition conditions studied here, not that calibration can generally be ignored.

#### 6.5.2. P1CH Components and Post-Processing

To assess the contribution of the main design choices, we evaluated P1CH variants by removing the residual blocks and by progressively applying post-processing to the raw pixel-wise predictions. The results are summarized in Table 8.

As shown in Table 8, removing the residual blocks reduces the accuracy to 95.76% and the macro F1 score to 88.73%, indicating that these blocks improve the stability of the learned spectral representation. The raw P1CH prediction already achieves strong performance; however, the median filter and the subsequent morphological processing further improve the results by reducing isolated pixel-level errors and refining border regions. Therefore, the combination of median filtering and morphology was retained as the final configuration used in this work.

#### 6.5.3. Robustness to Synthetic Illumination and Noise Perturbations

To further examine the sensitivity of P1CH to acquisition variability, synthetic intensity scaling and additive Gaussian noise were applied to the object-disjoint test spectra. The results are reported in Table 9.

The robustness results show that the model is tolerant to moderate intensity changes and noise, since the performance remains close to the unperturbed case under intensity scaling of ×0.8 and ×1.2, as well as under 30 dB noise. Larger degradation is observed for stronger perturbations, especially for intensity scaling of ×0.6 and 20 dB noise, where the macro F1 score decreases noticeably. This behavior is also consistent with the physics of hyperspectral imaging, where the measured spectral radiance depends on both the incident illumination spectrum and the material reflectance; therefore, stable and properly characterized illumination is required to recover reliable reflectance spectra and discriminate objects based on their spectral signatures [53]. These results confirm that wider illumination and color-temperature variation should be studied in future work using additional physical acquisitions.

### 6.6. Limitations

**Figure 14:**
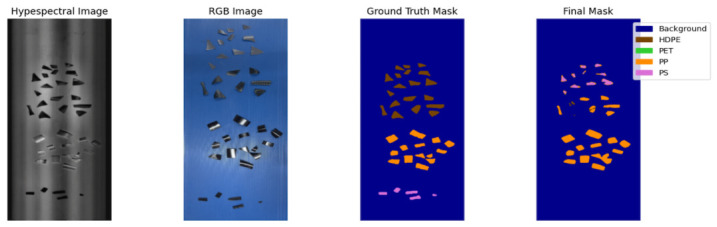
From left to right: The false-colour HS image, the RGB equivalent image, the Ground Truth mask, as well as the classification map generated by the P1CH classifier in the case of black plastics.

Despite the impressive results acquired in the previous experiments, this work intends to also underline the limitations of the HS imaging in classifying materials. To this end, the last experiment involves the analysis of dark-coloured, irregularly shaped objects. For the needs of this experiment, black and dark-coloured HDPE, PP and PS objects were cut in random small fractions and placed on top of the conveyor belt. The respective images and masks are presented in Figure 14. This subsection delves into the results of the analysis on dark objects. It also discusses the reasons why the model performs poorly in that case.

Based on an examination of the predictions in the last column of Figure 14, it is concluded that the model performs poorly in the case of black plastics. In detail, only PP samples were correctly classified, although lacking in mask precision. The PS samples were completely undetected, while some HDPE samples were detected but once again misclassified as PS or PP. The performance of P1CH in terms of accuracy, without taking into consideration the background, is equal to merely 39.69%.

**Figure 15:**
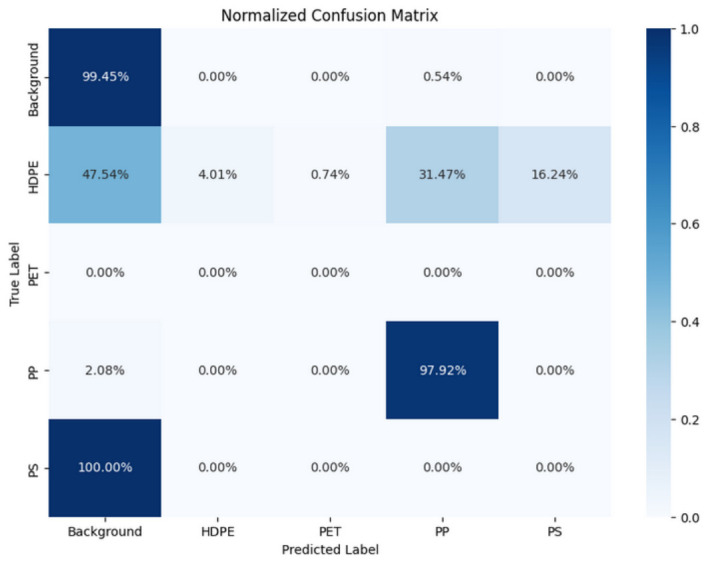
Normalized Confusion Matrix for the predictions of the model in the case of black and dark-coloured objects.

The confusion matrix presented in Figure 15 summarizes the models performance specifically in the case of black objects. In detail, it is confirmed that all the PS pixels were classified as background, i.e., they were not detected by the model at all. Moreover, this confusion matrix confirms that the PP samples were accurately classified, with the models precision in that case dropping to 75% since HDPE samples are also misclassified as PP. Finally, for the HDPE case, only 4.01% of the total samples were correctly classified, with the model’s recall for the specific case being equal to 46%.

**Figure 16:**
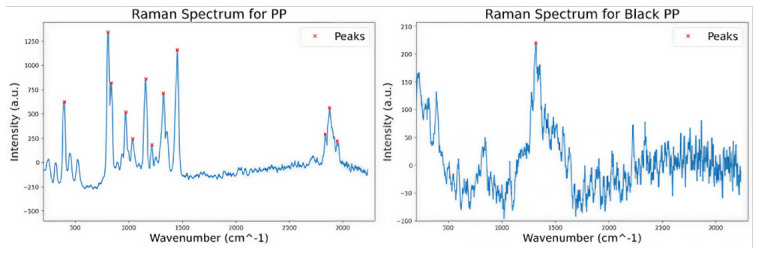
A comparison between the spectra acquired from a black (**left**) and a white (**right**) PS object. A significant drop in the signal’s amplitude can be observed in the case of black PS. Also one can notice that most of the indicative peaks for PS are non-existent in the case of black PS.

At this point, it is of paramount importance to explain the reasons that lead in this drop in the model’s performance. Specifically, this phenomenon should mainly be attributed to the nature of dark-coloured materials, rather than being considered a deficiency in the model. In detail, the observed dark or even black colour of an object is the result of complete or almost complete absorption of the incident radiation. In this manner, the reflected radiation, which is captured by the HS cameras’ sensor, is of very low intensity, hence resulting in a very weak digital signal. Therefore, given the low amplitude of the captured signal, the PSNR is consequently also low, and so is the variability of the spectrum. All of the above result in very similar—almost identical—noisy features of the model’s input vector, thus rendering the model incapable of properly analysing each pixel’s spectrum and ultimately correctly classifying them in their respective classes. An example of the spectrum of a black and a white plastic of the same material are presented in Figure 16. It can be easily observed that the spectrum of the black-coloured object is much more noisy with 10 times lower intensity.

It should also be highlighted that black plastics of any material class were absent from the training set. The poor performance on black plastics should therefore not be interpreted as a definitive limitation of all spectral learning approaches, but as a limitation of the present spectral range (i.e., 900–1700 nm), illumination geometry, exposure settings, and training distribution. Several directions may improve performance on dark materials: increasing exposure or sensor sensitivity, using cooled detectors, applying high-dynamic-range acquisition, changing illumination angle or polarization to reduce specular losses, using stronger or multi-angle active illumination, extending the spectral range beyond 1700 nm toward longer SWIR/MWIR bands, optimizing the conveyor/substrate background, and applying advanced denoising or spectral unmixing. The Raman measurements suggest that some dark samples may still contain weak but detectable material-specific peaks; therefore, hybrid Raman-HS sensing, peak-sensitive spectral models, and signal-to-noise management strategies are promising future research directions.

## 7. Conclusions & Future Work

In this paper, we proposed a lightweight, real-time *Pixel-wise 1D Convolutional Hyperspectral* (P1CH) classifier for material classification in hyperspectral images. The proposed model produces detailed and accurate classification maps and, unlike conventional RGB-based approaches, exploits the rich spectral information contained in hyperspectral data. This allows it to detect and classify objects even under challenging conditions where RGB imagery alone is often insufficient.

To train and validate the model, two hyperspectral image datasets were created. Both dataset splits include samples of *HDPE*, *PET*, *PP*, and *PS*. The training set consists of images containing objects from a single class only, whereas the test set was designed to reflect more demanding and realistic scenarios. In particular, it includes very small and irregularly shaped objects, overlapping items, and mixtures of materials with highly similar—and in some cases nearly identical—textures. In addition, this work introduces a simplified and cost-efficient spectral calibration and normalization procedure that does not rely on specialised hardware. The proposed model was evaluated on the object-disjoint test set and achieved an overall all-pixel accuracy of 97.44%, while when the pre-defined two-pixel boundary band was excluded in the sensitivity analysis, the accuracy increased to 99.94%. To further investigate the limitations of the P1CH classifier, an additional experiment on black objects was conducted, revealing that the model has difficulty classifying such samples reliably.

Overall, this work demonstrates the potential of pixel-wise hyperspectral imaging for material classification in conveyor-belt conditions, while also identifying limitations that require further study. The current 1D architecture is appropriate when individual pixels are dominated by one material and the classes have separable spectral signatures. Extending the approach to a wider range of polymers, non-plastics, multilayer packaging, additives, contaminated waste, or composite materials will benefit from larger and more diverse datasets, hierarchical or open-set classification, spectral unmixing for mixed pixels, and possibly multi-sensor fusion. Hybrid spatial–spectral architectures are also promising, provided that spatial information is incorporated in an edge-aware way that does not mix spectra across material boundaries. Future work will therefore focus on industrial illumination variability, black-plastic sensing, broader material taxonomies and domain adaptation.

## Figures and Tables

**Table 1:** Summary of Images, Objects, and Pixels per Category of the Training set.

Material	Images	Objects	Pixels
HDPE	2	40	794,142
PET	2	24	810,483
PP	2	45	831,340
PS	2	60	801,936

**Table 2:** Examples of object diversity included in the HS dataset.

Material	Representative Object Types	Colour/Opacity	Shape/Texture
HDPE	Detergent/shampoo bottles, bottle bodies, caps, rigid containers	White, light grey, dark-blue, yellow, green, light-blue, red, glossy and matte	Curved bottle bodies, flat fragments, lids, shredded pieces
PET	Transparent beverage bottles, trays, thin packaging pieces	Transparent, glossy and matte	Thin curved surfaces, bottle necks, flat pieces, small shards
PP	Bottle caps, food-container lids, yogurt/packaging components	White, red, blue, grey, green, opaque, semi-gloss	Rigid lids, curved caps, flat surfaces, irregular fragments
PS	Cups, plates, trays, foam-like and rigid PS pieces	White, glossy and matte	Flat trays, thin plates, brittle fragments, shredded pieces

**Table 3:** Black object test subset.

Material	Objects	Pixels
HDPE	19	31,649
PET	0	0
PP	14	25,280
PS	5	5035

**Table 4:** Test set without black objects.

Material	Objects	Pixels
HDPE	29	124,109
PET	20	100,445
PP	2	59,682
PS	29	116,704

**Table 5:** Class-wise test-set performance of P1CH on the object-disjoint test set.

Class	Precision (%)	Recall (%)	F1-Score (%)	Support (Pixels)
Background	99.35	97.82	98.58	3,096,015
HDPE	92.59	92.65	92.62	124,109
PET	83.07	96.09	89.11	100,445
PP	79.68	91.29	85.09	59,682
PS	80.70	96.76	88.00	116,704
**Overall/macro**	87.08	94.92	90.68	3,496,955

**Table 6:** Performance Metrics Comparison between P1CH and HybridSN Models.

Metric	P1CH	HybridSN
Mean Accuracy (%)	97.44	21.81
Mean Recall (%)	94.92	7.86
Mean Kappa Score	0.9295	−0.0795
Mean Inference Time (s)	5.06	34.29

**Table 7:** Effect of normalization strategy on object-disjoint test performance.

Preprocessing Strategy	Overall Accuracy (%)	Macro F1 (%)	Comment
Black/white reference normalization	97.95	91.56	Manufacturer-recommended reference procedure
Black reference + training maximum *M*	97.44	90.96	Proposed low-cost approximation used in this work
Black reference only	88.71	79.84	More sensitive to intensity scale variations

**Table 8:** Ablation study of P1CH components and post-processing.

Configuration	Accuracy (%)	Macro F1 (%)	Observation
P1CH without residual blocks	95.76	88.73	Lower spectral feature stability
P1CH raw prediction, no post-processing	96.86	89.95	Most errors are isolated pixels and borders
P1CH + median filter	97.27	90.42	Reduces isolated salt-and-pepper labels
P1CH + median + morphology	97.44	90.68	Final configuration used in this paper

**Table 9:** Robustness of P1CH to synthetic illumination and noise perturbations applied to the object-disjoint test spectra.

Perturbation	Accuracy (%)	Macro F1 (%)
No perturbation	97.44	90.68
Intensity scaling ×0.8	96.91	89.94
Intensity scaling ×1.2	97.08	90.11
Intensity scaling ×0.6	94.62	86.52
Additive Gaussian noise, SNR 30 dB	96.84	89.86
Additive Gaussian noise, SNR 20 dB	93.58	84.77

## Data Availability

The data presented in this study are available on request from the corresponding author.This is an ongoing experimental work, part of a Horizon Europe program. We are limited by the terms in the GA contract. Upon request, and approval, we will be able to share the dataset used in this work.
